# Understanding *Anaplasmataceae* pathogenesis using “Omics” approaches

**DOI:** 10.3389/fcimb.2014.00086

**Published:** 2014-07-02

**Authors:** Ludovic Pruneau, Amal Moumène, Damien F. Meyer, Isabel Marcelino, Thierry Lefrançois, Nathalie Vachiéry

**Affiliations:** ^1^CIRAD, BIOS, UMR CMAEEPetit-Bourg, France; ^2^INRA, BIOS, UMR CMAEEMontpellier, France; ^3^Université des Antilles et de la GuyanePointe-à-Pitre, France; ^4^IBETApartado, Oeiras, Portugal; ^5^ITQB-UNL, Estação Agronómica NacionalOeiras, Lisboa, Portugal

**Keywords:** *Anaplasmataceae*, *Anaplasma*, *Ehrlichia*, proteomics, transcriptomics, pathogenesis

## Abstract

This paper examines how “Omics” approaches improve our understanding of *Anaplasmataceae* pathogenesis, through a global and integrative strategy to identify genes and proteins involved in biochemical pathways key for pathogen-host-vector interactions. The *Anaplasmataceae* family comprises obligate intracellular bacteria mainly transmitted by arthropods. These bacteria are responsible for major human and animal endemic and emerging infectious diseases with important economic and public health impacts. In order to improve disease control strategies, it is essential to better understand their pathogenesis. Our work focused on four *Anaplasmataceae*, which cause important animal, human and zoonotic diseases: *Anaplasma marginale, A. phagocytophilum, Ehrlichia chaffeensis, and E. ruminantium*. *Wolbachia* spp. an endosymbiont of arthropods was also included in this review as a model of a non-pathogenic *Anaplasmataceae*. A gap analysis on “Omics” approaches on *Anaplasmataceae* was performed, which highlighted a lack of studies on the genes and proteins involved in the infection of hosts and vectors. Furthermore, most of the studies have been done on the pathogen itself, mainly on infectious free-living forms and rarely on intracellular forms. In order to perform a transcriptomic analysis of the intracellular stage of development, researchers developed methods to enrich bacterial transcripts from infected cells. These methods are described in this paper. Bacterial genes encoding outer membrane proteins, post-translational modifications, eukaryotic repeated motif proteins, proteins involved in osmotic and oxidative stress and hypothetical proteins have been identified to play a key role in *Anaplasmataceae* pathogenesis. Further investigations on the function of these outer membrane proteins and hypothetical proteins will be essential to confirm their role in the pathogenesis. Our work underlines the need for further studies in this domain and on host and vector responses to infection.

## Introduction

Understanding of *Anaplasmataceae* biology and pathogenesis has been greatly hampered by their obligatory intracellular characteristic, resulting in culture constraints and difficulties in studying the genetics of these bacteria. During the last 10 years, development of *in vitro* models and significant technical progress in molecular biology using high throughput methods allowed new knowledge to be garnered about their genome expression.

Herein, we review recent advances in the understanding of the *Anaplasmataceae* pathogenesis, using transcriptomics and proteomics approaches. First, we present the general characteristics of *Anaplasmataceae* and then focus on the most studied *Anaplasmataceae* bacteria using “Omics” approaches.

## *Anaplasmataceae* and their associated diseases

The *Anaplasmataceae* family belongs to the *Rickettsiales* order, which includes small obligate intracellular α-proteobacteria, most closely related to mitochondria (Merhej and Raoult, 2011). These bacteria are responsible for major endemic and emerging human and animal infectious diseases with important economic and public health impacts. The *Anaplasmataceae* family includes six genera, *Ehrlichia, Anaplasma, Aegyptianella, Wolbachia, Neorickettsia* and “*Candidatus* Neoehrlichia” (Dunning Hotopp et al., 2006). These bacteria infect invertebrate hosts that are abundant and ubiquitous in the environment (i.e., ticks, insects, trematodes, nematodes, or mollusks). *Neorickettsia* and *Wolbachia* spp. can be transmitted through generations of invertebrate hosts by both transovarial and trans-stadial transmission, whereas *Anaplasma* and *Ehrlichia* seem to have only trans-stadial transmission (Stich et al., 1989; Long et al., 2003). All genera except *Wolbachia* are known to infect vertebrates (mammals or birds). The bacteria infect specific host cell types, such as neutrophils, monocytes and macrophages, platelets, erythrocytes or endothelial cells depending on the species. Within the *Anaplasmataceae* family, *Anaplasma phagocytophilum* and *Ehrlichia chaffeensis*, which infect humans, have been widely studied.

Table 1 shows host, vector and geographical distributions for *A. marginale, A. phagocytophilum, E. chaffeensis, E. ruminantium*, and *Wolbachia* spp. *A. phagocytophilum* is responsible for anaplasmosis and infects deer, dogs, cats, horses, ruminants, rodents, and humans, inducing human granulocytic anaplasmosis. *E. chaffeensis* infects deer, dogs, and humans, inducing canine ehrlichiosis and human monocytic ehrlichiosis; whereas *A. marginale* and *E. ruminantium* infect only domestic and wild ruminants. *A. marginale* and *E. ruminantium* cause bovine anaplasmosis and heartwater, respectively. Bovine anaplasmosis has the widest distribution among tick-borne diseases. The acute form of the disease induces fever, anemia, weight loss and often death. After infection, animals are asymptomatic carriers and constitute a reservoir for the transmission of the disease. Heartwater is present in Sub-Saharan Africa, the Caribbean and Indian Ocean islands and induces mortality in ruminants and decreases herd productivity. The economic impact of heartwater, associated to mortality and cost treatment (antibiotic and acaricide), is estimated as $46.7 million per year for the Southern community (Vachiéry et al., 2013).

**Table 1:** **Hosts, vectors, geographical distribution, and associated diseases for selected *Anaplasmataceae* spp**.

**Species**	**Host**	**Principal vector**	**Disease**	**Geographical distribution**
*A. phagocytophilum*	Humans, deer, dogs, cats, horses, ruminants, rodents	Ticks *Ixodes* spp.	Human granulocytic anaplasmosis, tick-born fever of ruminants, anaplasmosis	USA, Europe, and Asia
*A. marginale*	Cattle, wild ruminants	Ticks *Rhicephalus microplus, Dermacentor*	Bovine anaplasmosis	Worldwide in tropical and subtropical regions
*E. chaffeensis*	Human, deer, dogs	Ticks *Amblyomma americanum*	Human monocytic ehrlichiosis, canine ehrlichiosis	USA, South America, and Asia
*E. ruminantium*	Cattle, sheep, goats, wild ruminants	Ticks *African Amblyomma* sp. *including variegatum* and *hebraeum*	Heartwater	Sub Saharan Africa, Comoros, Mayotte, Reunion island, Madagascar, Caribbean
*Wolbachia* spp.	Nematod *Brugia malayi*, Arthropod *Anopheles gambiae*	NA	NA	Worldwide distribution

**Figure 1:**
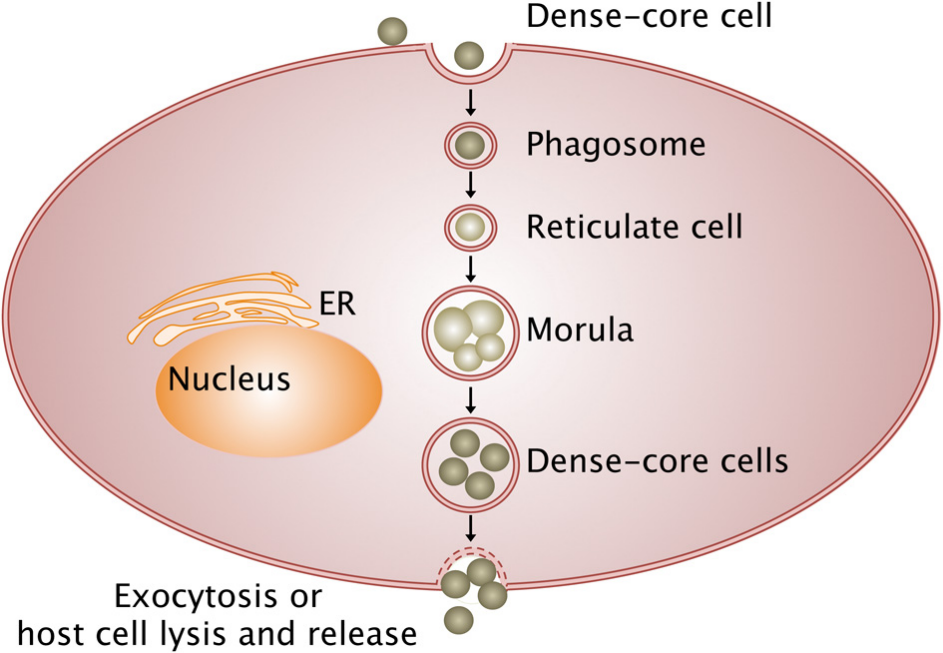
**Intracellular life cycle of *Anaplasmataceae***. The *Anaplasmataceae* dense-cored cell develops into replicating reticulate cells in the phagosome, which does not fuse with lysosome and forms morulae (vegetative form). Then, reticulate cells mature into dense-cored cells that are liberated by exocytosis or host cell lysis (infectious form). Arrows, transition between stages; ER, endoplasmic reticulum.

Bacteria from the *Anaplasmataceae* family develop within a cytoplasmic vacuole in the host cell cytoplasm, whereas other members of *Rickettsiales* order escape from the phagosome after entering the host cell and multiply in the cytoplasm before being released in the extracellular environment (Figure 1). Different stages of development are defined for *Anaplasmataceae*, which are characterized by their DNA reorganization from dense cored cell (infectious form) to reticulate cell (vegetative form). The reticulated forms multiply by binary fission and form morulae, and then turn into the dense cored cells before being released.

## General features of transcriptomic and proteomic studies

Transcriptomics and proteomics describe the complete, or nearly complete, collection of transcripts and proteins of an organism (bacterium, host, or vector) in different conditions. These conditions include the bacterium growth in different arthropod or vertebrate host cells and tissues. Careful analysis of the transcriptome and proteome is essential to understand the functional output of the genome of bacteria, hosts, and vectors (Filiatrault, 2011).

More specifically, in addition to comparative genomics, transcriptomics and proteomics constitute powerful approaches to increase our knowledge on *Anaplasmataceae* pathogenesis. Researchers have the opportunity to study both sides: (i) the functional genomics of *Anaplasmataceae* in host or vector cells and (ii) the functional genomics of host or vector in response to *Anaplasmataceae* infection.

**Figure 2:**
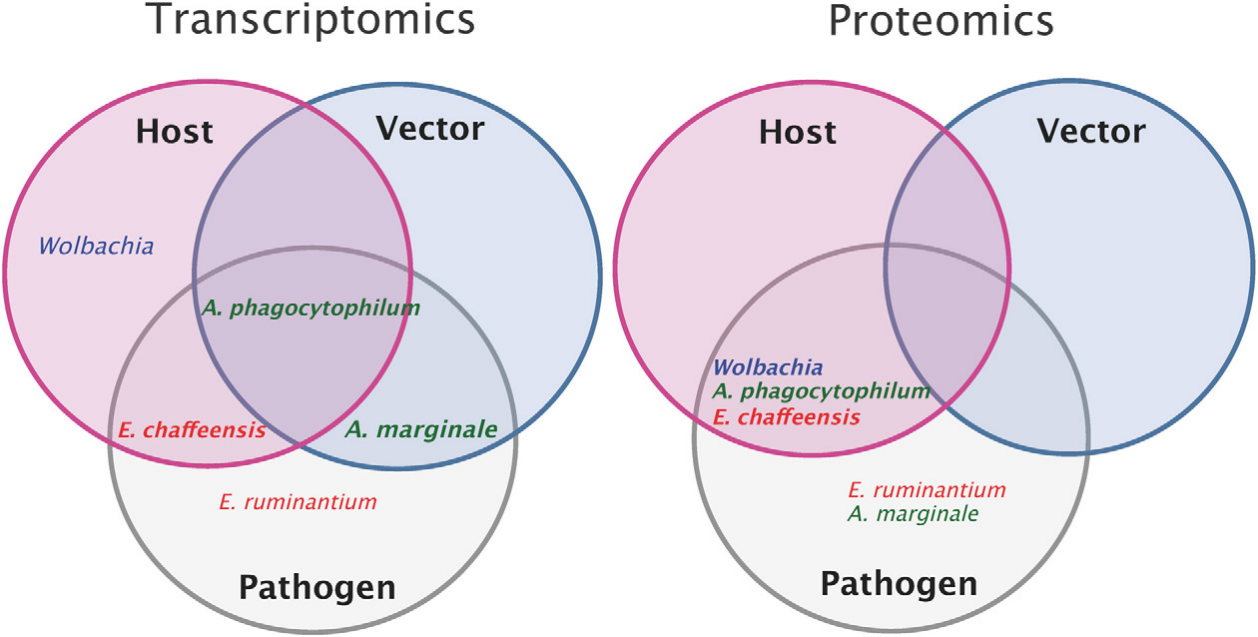
**Overview of pathogen, host, and vector transcriptomic and proteomic studies**. Transcriptomic or proteomic studies done on host (pink circle) and on vector (blue circle) after pathogen infection (the name of the pathogen is indicated for each study). Transcriptomic or proteomic studies done on the pathogen (gray). Intersections of circles correspond to studies done both on the pathogen and on host or/and vector. In example, transcriptomic studies of *Anaplasma phagocytophilum* and of its host and vector have been done whereas only proteomic studies of *Anaplasma phagocytophilum* and its host have been performed.

An overview of the different transcriptomic and proteomic studies currently available on the 5 different *Anaplasmataceae* is represented in Figure 2. A complete analysis of host and vector transcriptomes in response to bacterial infection as well as the pathogen transcriptome has only been done for *A*. *phagocytophilum*. Additionally, the pathogen and host proteomes are also available. For *E. ruminantium*, transcriptomic and proteomic studies were performed only on the pathogen side and there are no data on host or vector responses to pathogen infection. Additionally, concerning the bacteria, proteomic and transcriptomic studies were done also on *E. chaffeensis* and *A. marginale*. The use of such “Omics” approaches on bacteria is particularly useful to perform integrative analyses, linking gene and protein expression for these 4 *Anaplasmataceae* (*E. ruminantium, E. chaffeensis, A. phagocytophilum*, and *A. marginale)*. There is a lack of data concerning the proteome of the vector after infection. The effect of *Wolbachia* on its host genes and protein expressions was studied in order to improve our comprehension of this symbiotic interaction, using *Anopheles gambiae* as model.

Globally, most of the functional genomic studies were performed on the pathogen and only preliminary studies on the host or vector sides are available. Thus, there is a strong need to study both vector and host transcriptome and proteome in response to infection in order to better understand pathogen-host and pathogen-vector interactions.

## Strategies and methods used to perform transcriptomic and proteomic studies on *Anaplasmataceae*

Various strategies are used to study *Anaplasmataceae* transcriptomic and proteomic studies. Authors generally compare the transcriptome and/or the proteome of (i) bacteria infecting different host and/or vector cells, (ii) bacterial strains with a distinct virulence level and/or from geographically distinct regions, and (iii) bacteria in *in vitro, in vivo*, and *ex vivo* conditions.

Until now, few transcriptomic studies have been performed on the different stages of development of *Anaplasmataceae* members, mainly due to technical constraints associated with the intracellular properties of the bacteria. In one of these studies, the authors compared the *in vitro* gene expression profile between *E. ruminantium* elementary bodies (extracellular dense-cored form) and reticulate bodies (intra-cellular non-infectious forms) (Pruneau et al., 2012). For *Wolbachia* spp., *E. chaffeensis, E. ruminantium, A. marginale*, and *A*. *phagocytophilum* transcriptomic and proteomic studies were performed in vertebrate host cells (*in vitro, in vivo*, or *ex vivo*). Additional studies were done on the pathogens grown in tick cells for *A. phagocytophilum* and *E. chaffeensis* (transcriptome) and for *E. chaffeensis, A. phagocytophilum*, and *A. marginale* (proteome). These different studies allowed further investigation of the interaction between bacteria and its host or vector and the identification of genes, essential for adaptation in host and vector cells.

One of the major constraints in studying the transcriptome and proteome of obligate intracellular bacteria is the large excess of mRNA and proteins of host or vector origin. The removal of eukaryotic RNA and prokaryotic rRNA contaminants is a prerequisite step for *Anaplasmataceae* transcriptomic studies. The depletion of eukaryotic RNA contaminant was achieved mostly through the use of substractive hybridization technologies. The classical approach developed by Ambion (MicrobEnrich Kit), consists of capturing oligonucleotides coupled with magnetic beads that hybridize to the 18S and 28S ribosomal RNA and the poly-adenylated 3′ tail of eukaryotic mRNA. An additional method, known as MicrobExpress (Ambion), uses similar magnetic beads and allows the enrichment of bacterial mRNA by capturing ribosomal and transfer RNA (La et al., 2007). More recently, Emboule and co-workers developed an alternative method for *E. ruminantium*. This method allows both the removal of the eukaryotic RNA and prokaryotic rRNA contaminants and mRNA enrichment from *E. ruminantium* origin (Emboule et al., 2009) and could be adapted for other *Anaplasmataceae*.

For proteomics, various methods have been used to enrich the amount of intracellular bacteria proteins. For example, biochemical fractionation based on differential density and size, or based on harsh detergent treatments dissolving differentially host cells, fractionation based on flow cytometry sorting, or a combination of any of these methods has been used. Purity and yield of the enriched bacteria samples can vary; but generally, dozens to hundreds of bacteria proteins could be successfully detected (Marcelino et al., 2012).

## Transcriptomes and proteomes of *Anaplasmataceae*

Functional genomics studies of *Anaplasmataceae* are essential to identify the main groups of genes regulated during infection of the host or vector. According to their Clusters of Orthologous genes (COG), these genes are classified into the following categories: cell wall membrane biogenesis (including outer membrane proteins); translation, replication and post-translational modifications (PTM); amino acids biosynthesis, metabolism and transport; energy production; intracellular trafficking and secretion and genes encoding enzymes counteracting osmotic and oxidative stress; and the largest category comprising genes encoding hypothetical proteins.

### Expression of outer membrane proteins (OMP) during *Anaplasmataceae* infection

OMPs play several important roles in bacteria, allowing them to adapt to different environments and host niches. These roles include biogenesis and integrity of the outer membrane, non-specific porin activity, adherence, and membrane associated enzymatic activity (Lin et al., 2002). Some OMPs are porins that form channels, allowing the transport of molecules across lipid bilayer membranes and play a major role in host-interaction. They contribute to nutrient transport, antimicrobial resistance and response to osmotic stress, and are essential for bacteria (Achouak et al., 2001).

OMPs bacteria seem to be crucial in obligate intracellular and could facilitate early interactions with the host and vector cells. They are detected in the proteome of the 5 *Anaplasmataceae* studied, and are the dominant proteins detected in the global proteome of these bacteria. For example, among the 113 *p44* paralogous genes in *A. phagocytophilum*, 110 of them were expressed in the human promyelocytic leukemia cell line, HL-60 (Lin et al., 2011). The first proteome of *E. ruminantium* elementary bodies showed that MAP1 protein was found to be the most predominant protein and seems to be organized as a porin (Marcelino et al., 2012). Other OMP such as MAP 1-6, MAP 1-14, and MAP 1+1 were also identified. Pruneau and co-workers showed an up-regulation of *map1-6* gene expression in *E. ruminantium* reticulate bodies (intracellular forms) compared to elementary bodies (free-living form) (Pruneau et al., 2012). The MAP protein family seems to be essential for *E. ruminantium* intracellular survival; however, their functions remain unknown until now and should be investigated.

### Differential expression of OMP in infected tick and host cells

In *A. phagocytophilum*, the locus p44, which encodes for several OMPs was expressed in human cell lines and not in tick cell lines. Furthermore, the authors showed a higher proportion of up-regulated genes encoding other OMPs in *A. phagocytophilum* infecting human cell lines rather than in tick cell lines (Nelson et al., 2008). For *E. ruminantium*, MAP 1-1 was expressed only in tick cells and not in host cells (Postigo et al., 2008). A differential expression of OMP-family genes between host cells and vector cells was also observed for *E. chaffeensis* (Kuriakose et al., 2011). These data showed that the OMPs of *Anaplasmataceae* are strongly regulated depending on host or vector cell types and could be important for the development within host and vector.

It would be interesting to better understand the role of these OMPs, which are at the interface between the pathogen, the host, and vector and can represent potential vaccines.

### Differential expression of genes involved in PTM of bacterial proteins in infected tick and host cells

*Anaplasmataceae* with up-regulation of genes leading to PTM can modulate host stress responses and escape from immune system recognition. For *E. chaffeensis*, Kuriakose and co-workers found genes involved in PTM, differentially expressed between host and vector cells, and showed a post-transcriptional regulation of certain *E. chaffeensis* genes involved in host-pathogen-vector interactions (Kuriakose et al., 2011). It would be interesting to further study PTMs on bacterial proteins due to their potential impact on pathogenesis.

### Differential expression of type IV secretion systems (T4SS) during *Anaplasmataceae* infection

All *Anaplasmataceae* have a T4SS, which consists of a multiprotein complex that injects effector proteins into eukaryotic cells. The paradigm of T4SS is that of *Agrobacterium tumefaciens*, which contains 12 *virB/D* genes. Except for *virB1* and *virB5*, all the components of the *A. tumefaciens* T4SS are conserved in *Anaplasmataceae*, but can be duplicated and scattered in several gene clusters (Gillespie et al., 2010). The crucial role of T4SS and its effector proteins in pathogenesis has already been shown for *A. phagocytophilum* and *E. chaffeensis* (Rikihisa and Lin, 2010).

In a recent study, global proteomes of *A. phagocytophilum* and *E. chaffeensis* in human promyelocytic leukemia cell line, HL-60, were characterized (Lin et al., 2011). Proteins of T4SS such as VirB4 and VirD4 were detected as well as the T4SS effectors, AnkA and Ats-1. T4SS components, namely VirB9 (basal body) and VirB11 (ATPase), were detected by proteomic analysis of *E. ruminantium* (Marcelino et al., 2012). For *A. phagocytophilum*, the up-regulation of T4SS genes had already been demonstrated in 3 cell lines: in human cells (HL-60 and HMEC-1) and in tick cells (ISE6) (Nelson et al., 2008). Surprisingly, the *virB2* paralogs were differentially transcribed between the human and tick cells. This result can reflect a specific use of T4SS components depending on the host. Moreover, the effector AnkA was strongly transcribed in HMEC-1, less transcribed in HL-60, and only marginally transcribed in ISE6 (Nelson et al., 2008). In another transcriptomic study, *ankA* was also identified by RNA-sequencing in *Ixodes scapularis* tick salivary glands infected with *A. phagocytophilum* (Mastronunzio et al., 2012). The AnkA effector protein modulates the expression of some host genes and seems to be crucial for *A. phagocytophilum* infection in host cells (Lin et al., 2007). The recent development of bioinformatics software for the identification of candidate T4SS effectors should facilitate the characterization of the role of these proteins of virulence in *Anaplasmataceae* pathogenesis (Meyer et al., 2013). Several components of T4SS were also detected in the global proteome of endosymbiont *Wolbachia* (Bennuru et al., 2011). This suggests a potential role of the T4SS during the endosymbiotic interaction.

### Expression of bacterial proteins to curb osmotic and oxidative host response

For *E. ruminantium*, genes encoding thioredoxin, *trx*, were found to be over-expressed at the reticulate body stage compared to the infectious free stage (Pruneau et al., 2012). Oxidative stress is part of innate immune response and results in a production of reactive oxygen species (ROS) from host cells, which degrade the bacterial membrane. The induction of anti-oxidant enzymes such as thioredoxin or superoxide dismutase diminishes the ROS activity. Other genes involved in the defense against oxidative stress were also up-regulated for *E. chaffeensis* (ECH_0493 encodes for superoxide dismutase) infecting mammalian cells as compared with infected vector cells (Kuriakose et al., 2011) and for *A. phagocytophilum* (APH_0795 encodes for antioxidant AhpC/Tsa family) (Nelson et al., 2008). Superoxide dismutase was also detected in the global proteome of *E. chaffeensis* (Seo et al., 2008). In *E. ruminantium* proteome, TsaA and ElbB proteins, both involved in cell redox homeostasis, were detected. Interestingly, among the several bacterial species compared, the authors found that ElbB was exclusively detected in elementary bodies of *E. ruminantium* (Marcelino et al., 2012). In host and vector cells, *Anaplasmataceae* also fight against osmotic stress by up-regulation of proline-betaine transporter (*proP*). Proline and betaine are two osmoprotectants, which help reduce the hyperosmolarity. These results, showing the fight against the first defense mechanisms of cells, reflect the successful adaptation of *Anaplasmataceae* to their host cells.

### Identification of pathogen metabolic activities

Functional genomics of the five *Anaplasmataceae*, focus of this review, revealed the presence of genes/proteins involved in metabolic pathways in host and vector cells. Indeed, many over-expressed genes or proteins were involved either in (i) energy production and conversion and (ii) the transport and metabolism of nucleotides, amino acids, inorganic ions, carbohydrates, and coenzymes.

For *E. ruminantium*, the transcriptome study comparing different stages of development showed that genes involved in the carbohydrate, amino acid, inorganic ion, nucleotide, and coenzyme transports and metabolisms were differentially expressed at both reticulate and elementary body stages. These results suggest that elementary bodies of *E. ruminantium* could be metabolically active (Pruneau et al., 2012). Moreover, at *E. ruminantium* elementary body stage, the majority of proteins detected were related to energy and general metabolism (Marcelino et al., 2012). Lin and co-workers analyzed the global proteome of *A. phagocytophilum* and *E. chaffeensis*. They identified many proteins involved in nucleotide, vitamin, and cofactor biosynthetic pathways (Lin et al., 2011). The comparison between *E. chaffeensis* transcriptome in human cells and in tick cells revealed a larger number of genes with high expression levels in the tick cells. These genes encode for proteins involved in energy production and conversion, nutrient transport, metabolism, cellular process, and translation. The up-regulation of these genes reveals that *E. chaffeensis* has higher metabolic activity in the vector cells than in mammalian cells (Kuriakose et al., 2011).

### Identification of important hypothetical proteins in the pathogenesis

Regardless of the adopted strategy in transcriptome or proteome studies, genes encoding hypothetical proteins were one of the most represented COG. Kuriakose and co-workers compared the *E. chaffeensis* transcriptome in mammalian *vs*. arthropod hosts (Kuriakose et al., 2011). They showed that genes encoding hypothetical proteins were the most up-regulated genes in human and tick cells. Furthermore, among these genes, they identified 11 highly expressed in human cells that were not expressed in tick cells and 18 expressed in tick cells and not in human cells. These genes do not have any orthologs in other *Ehrlichia* spp. and seem to be required for adaptation and survival in host and vector cells (Kuriakose et al., 2011). Further studies are needed to characterize the function of these genes encoding hypothetical proteins and their role in adaptation and survival in host or vector. Recently, two studies allowed the characterization of the function of the hypothetical protein APH_1235 in *A. phagocytophilum*. The gene encoding APH_1235 was found to be up-regulated in dense cored form (Troese et al., 2011) and the blocking of protein APH_1235 with antibodies reduced infection levels in mammalian cells (Mastronunzio et al., 2012). This result indicates that APH_1235 is required for host cell infection. This protein has homologs in other *Anaplasma* spp. and *Ehrlichia* spp. but not in other bacteria. In *E. ruminantium*, CDS_00640, ortholog of APH_1235, was also up-regulated in the infectious elementary bodies similar to the dense cored form in *A. phagocytophilum* (Pruneau et al., 2012). Even if gene homology analysis is the first step to characterize unknown genes, further experiments will be useful to validate the role of this gene in host cell infection by *E. ruminantium*.

## Transcriptome and proteome of infected host and ticks

### Functional genomics of host cells in response to infection

Regarding functional genomics of host cells in response to infection with *Anaplasmataceae* members, up-regulated genes are generally involved in defense mechanisms and immune responses, such as genes encoding interferon, cytokines, chemokines, and their receptors. Many of these genes were over-expressed in host cells infected with *A. phagocytophilum* (Lee et al., 2008) and *E. chaffeensis* (Miura and Rikihisa, 2009). These genes encode for proteins essential in the first line of defense against bacteria. For *A. phagocytophilum*, the up-regulation of these genes was observed at the early stages post-infection, most of them being down-regulated at later stages post-infection. It seems that *A. phagocytophilum* modulates the expression of these genes during infection to survive in host cells (Lee et al., 2008). In another study, gene expression analyses, using microarrays and real time RT-PCR, were performed in naturally and experimentally infected pigs with *A. phagocytophilum*. These analyses revealed the up-regulation of immune response genes, such as interleukin 1 receptor accessory protein-like 1 (*IL1RAPL1*), T-cell receptor alpha chain (*TCR-alpha*), thrombospondin 4 (*TSP-4*), and Gap junction protein alpha 1 (*GJA1*) genes. These results suggest that pigs control the bacterial infection, particularly through activation of innate immune responses, phagocytosis, and autophagy (Galindo et al., 2012).

Apoptotic pathways are inhibited due to the up-regulation of anti-apoptotic genes and down-regulation of pro-apoptotic genes. This was observed for *A. phagocytophilum* (Lee et al., 2008) and *E. chaffeensis* (Miura and Rikihisa, 2009).

In the study by Miura and Rikihisa, liver cell transcriptome was studied in response to infection with the three strains of *E. chaffeensis*: Wakulla, Liberty, and Arkansas, which induce different histopathologic lesions in liver tissue. The expression profiles of cytokines, chemokines, and their receptors were found to be different among the three strains and could be therefore related to the distinct histopathological lesions (Miura and Rikihisa, 2009).

### Functional genomics of vector cells in response to infection

Concerning vector response to *Anaplasmataceae* infection, only 3 transcriptomics studies have been performed. In the first study, the authors compared *Ixodes scapularis* transcriptome in response to infection with *A. phagocytophilum* and *A. marginale* (Zivkovic et al., 2009). Gene expression profiles were found to be different between the 2 *Anaplasma* species. This difference may reflect the difference of *Anaplasma* spp. developmental cycle in tick cells, or may be due to the fact that *I. scapularis* is not a natural vector of *A. marginale* (Zivkovic et al., 2009). In the second and third studies, the authors studied the transcriptome of vector *Rhipicephalus microplus* infected with *A. marginale* (Zivkovic et al., 2010; Mercado-Curiel et al., 2011). They showed that few genes were regulated in *R. microplus* salivary glands after infection with *A. marginale* (Zivkovic et al., 2010). There was a limited impact of *A. marginale* infection on the tick gene expression compared to uninfected ticks, suggesting minor effects on tick activity or survival. The differentially expressed genes encoding putative proteins are involved in binding, catalytic/enzymatic activity, transport, DNA/RNA metabolism, and structural molecules. Among them, three genes putative von Willebrand factor, a flagelliform silk protein, and subolesin genes, seem to be important for infection and multiplication of *A. marginale* in *R. microplus*. In the third study, the authors showed a differential gene expression between the *R. microplus* midgut and salivary gland in response to feeding and regulation in the salivary gland over a period of time (Mercado-Curiel et al., 2011). This result illustrates the need to study the pathogen-vector interaction during the feeding process.

## Conclusion and future perspectives

*Anaplasmataceae* organisms must be very versatile to survive in different microenvironments. During their extracellular and infective stage, they need to escape the immune system. Inside the host/vector cells, they need to counteract the innate immune response and to subvert cellular processes to survive and/or replicate. Functional genomics facilitate the study of the modulation of genes and proteins expression depending on environmental conditions. A first high-throughput screening of gene/protein expression by “Omics” approaches on the pathogen/host/vector allows a general view of differentially expressed genes/proteins. The second step involves the focusing on specific mechanisms, gene function and pathways of interest to go further in the comprehension of the pathogen biology and pathogenesis using classical approaches.

Resistance to host innate defense mechanisms, like osmotic stress and oxidative burst, are identified in several studies and these mechanisms seem to play a key role for the *Anaplasmataceae* intracellular development. In addition, OMPs and PTMs seem to have an important role in the pathogenesis.

The majority of transcripts and proteins identified by these studies are of unknown functions. They probably have an important role in pathogenesis and should be investigated. The generation of knockout mutants for genes of interest is now technically feasible in *Anaplasmataceae*. The recent development of transient and stable *in vitro* transfection systems for *A. marginale* (Felsheim et al., 2010; Noh et al., 2011) and the optimization of random and targeted mutagenesis for *E. chaffeensis* (Cheng et al., 2013) pave the way for further promising functional analyses of these bacteria. For example, in a recent study, the authors compared the global transcriptome of transformed *A. marginale* strain *vs*. wild type and identified candidate genes for the development of slow growing attenuated vaccines (Pierle et al., 2013). The development of reliable high throughput RNA sequencing methods will replace the use of microarrays for the better understanding of bacterial biology. This could lead to the deciphering of the role of non-coding RNAs, which have not been studied in *Anaplasmataceae*. They could be involved in the regulation of key processes and could have a major role in the pathogenesis of these bacteria.

In the past decade, significant efforts in improving analytical technologies related to measuring mRNA, proteins, and metabolites have been made. Nowadays, new breakthroughs in host-pathogen-vector research rely on the development of novel “Omics” approaches that incorporate high throughput sequencing or separation technologies, and other less known “Omics” such as metabolomics, immunomics, and vaccinomics (Bagnoli et al., 2011). In the future, integrated “Omics” investigation of various cellular molecules and their interaction in cells (i.e., interactomes) could lead to a quantified description of cellular metabolism for further hypothesis-driven investigation. Those efforts will probably lead to fundamentally new insights into bacterial metabolism during intracellular development.

## Author contributions

Ludovic Pruneau: Gathering information and papers and writing the paper; Nathalie Vachiéry: Proposing the subject and writing the paper; Amal Moumène, Damien F. Meyer: Preparation and interpretation of the figures, gathering information and reviewing the paper. Thierry Lefrançois and Isabel Marcelino: reviewing the paper. All authors agreed on outlines and the final version of the paper.

### Conflict of interest statement

The authors declare that the research was conducted in the absence of any commercial or financial relationships that could be construed as a potential conflict of interest.

## References

[B1] Achouak W. Heulin T. Pages J. M. (2001). Multiple facets of bacterial porins. FEMS Microbiol. Lett. 199, 1–7 10.1111/j.1574-6968.2001.tb10642.x 11356559

[B2] Bagnoli F. Baudner B. Mishra R. P. Bartolini E. Fiaschi L. Mariotti P. (2011). Designing the next generation of vaccines for global public health. OMICS 15, 545–566 10.1089/omi.2010.0127 21682594

[B3] Bennuru S. Meng Z. Ribeiro J. M. Semnani R. T. Ghedin E. Chan K. (2011). Stage-specific proteomic expression patterns of the human filarial parasite *Brugia malayi* and its endosymbiont *Wolbachia*. Proc. Natl. Acad. Sci. U.S.A. 108, 9649–9654 10.1073/pnas.1011481108 21606368 PMC3111283

[B4] Cheng C. Nair A. D. Indukuri V. V. Gong S. Felsheim R. F. Jaworski D. (2013). Targeted and random mutagenesis of *Ehrlichia chaffeensis* for the identification of genes required for *in vivo* infection. PLoS Pathog. 9:e1003171 10.1371/journal.ppat.1003171 23459099 PMC3573109

[B5] Dunning Hotopp J. C. Lin M. Madupu R. Crabtree J. Angiuoli S. V. Eisen J. A. (2006). Comparative genomics of emerging human ehrlichiosis agents. PLoS Genet. 2:e21 10.1371/journal.pgen.0020021 16482227 PMC1366493

[B6] Emboule L. Daigle F. Meyer D. F. Mari B. Pinarello V. Sheikboudou C. (2009). Innovative approach for transcriptomic analysis of obligate intracellular pathogen: selective capture of transcribed sequences of *Ehrlichia ruminantium*. BMC Mol. Biol. 10:111 10.1186/1471-2199-10-111 20034374 PMC2806407

[B7] Felsheim R. F. Chavez A. S. Palmer G. H. Crosby L. Barbet A. F. Kurtti T. J. (2010). Transformation of *Anaplasma marginale*. Vet. Parasitol. 167, 167–174 10.1016/j.vetpar.2009.09.018 19837516 PMC2817780

[B8] Filiatrault M. J. (2011). Progress in prokaryotic transcriptomics. Curr. Opin. Microbiol. 14, 579–586 10.1016/j.mib.2011.07.023 21839669

[B9] Galindo R. C. Ayllon N. Smrdel K. S. Boadella M. Beltran-Beck B. Mazariegos M. (2012). Gene expression profile suggests that pigs (*Sus scrofa*) are susceptible to *Anaplasma phagocytophilum* but control infection. Parasit. Vectors 5, 181 10.1186/1756-3305-5-181 22935149 PMC3453518

[B10] Gillespie J. J. Brayton K. A. Williams K. P. Diaz M. A. Brown W. C. Azad A. F. (2010). Phylogenomics reveals a diverse *Rickettsiales* type IV secretion system. Infect. Immun. 78, 1809–1823 10.1128/IAI.01384-09 20176788 PMC2863512

[B11] Kuriakose J. A. Miyashiro S. Luo T. Zhu B. McBride J. W. (2011). *Ehrlichia chaffeensis* transcriptome in mammalian and arthropod hosts reveals differential gene expression and post transcriptional regulation. PLoS ONE 6:e24136 10.1371/journal.pone.0024136 21915290 PMC3167834

[B12] La M. V. Francois P. Rovery C. Robineau S. Barbry P. Schrenzel J. (2007). Development of a method for recovering *rickettsial* RNA from infected cells to analyze gene expression profiling of obligate intracellular bacteria. J. Microbiol. Methods 71, 292–297 10.1016/j.mimet.2007.09.017 17964675

[B13] Lee H. C. Kioi M. Han J. Puri R. K. Goodman J. L. (2008). *Anaplasma phagocytophilum*-induced gene expression in both human neutrophils and HL-60 cells. Genomics 92, 144–151 10.1016/j.ygeno.2008.05.005 18603403

[B14] Lin J. Huang S. Zhang Q. (2002). Outer membrane proteins: key players for bacterial adaptation in host niches. Microbes Infect. 4, 325–331 10.1016/S1286-4579(02)01545-9 11909743

[B15] Lin M. den Dulk-Ras A. Hooykaas P. J. Rikihisa Y. (2007). *Anaplasma phagocytophilum* AnkA secreted by type IV secretion system is tyrosine phosphorylated by Abl-1 to facilitate infection. Cell. Microbiol. 9, 2644–2657 10.1111/j.1462-5822.2007.00985.x 17587335

[B16] Lin M. Kikuchi T. Brewer H. M. Norbeck A. D. Rikihisa Y. (2011). Global proteomic analysis of two tick-borne emerging zoonotic agents: *Anaplasma phagocytophilum* and *Ehrlichia chaffeensis*. Front. Microbiol. 2:24 10.3389/fmicb.2011.00024 21687416 PMC3109344

[B17] Long S. W. Zhang X. Zhang J. Ruble R. P. Teel P. Yu X. J. (2003). Evaluation of transovarial transmission and transmissibility of Ehrlichia chaffeensis (*Rickettsiales: Anaplasmataceae*) in *Amblyomma americanum* (*Acari: Ixodidae*). J. Med. Entomol. 40, 1000–1004 10.1603/0022-2585-40.6.1000 14765684

[B18] Marcelino I. de Almeida A. M. Brito C. Meyer D. F. Barreto M. Sheikboudou C. (2012). Proteomic analyses of *Ehrlichia ruminantium* highlight differential expression of MAP1-family proteins. Vet. Microbiol. 156, 305–314 10.1016/j.vetmic.2011.11.022 22204792

[B19] Mastronunzio J. E. Kurscheid S. Fikrig E. (2012). Postgenomic analyses reveal development of infectious *Anaplasma phagocytophilum* during transmission from ticks to mice. J. Bacteriol. 194, 2238–2247 10.1128/JB.06791-11 22389475 PMC3347074

[B20] Mercado-Curiel R. F. Palmer G. H. Guerrero F. D. Brayton K. A. (2011). Temporal characterisation of the organ-specific *Rhipicephalus microplus* transcriptional response to *Anaplasma marginale* infection. Int. J. Parasitol. 41, 851–860 10.1016/j.ijpara.2011.03.003 21514300 PMC3114074

[B21] Merhej V. Raoult D. (2011). *Rickettsial* evolution in the light of comparative genomics. Biol. Rev. Camb. Philos. Soc. 86, 379–405 10.1111/j.1469-185X.2010.00151.x 20716256

[B22] Meyer D. F. Noroy C. Mouméne A. Raffaele S. Albina E. Vachiéry N. (2013). Searching algorithm for type IV secretion system effectors 1.0: a tool for predicting type IV effectors and exploring their genomic context. Nucleic Acids Res. 41, 9218–9229 10.1093/nar/gkt718 23945940 PMC3814349

[B23] Miura K. Rikihisa Y. (2009). Liver transcriptome profiles associated with strain-specific *Ehrlichia chaffeensis*-induced hepatitis in SCID mice. Infect. Immun. 77, 245–254 10.1128/IAI.00979-08 19001077 PMC2612275

[B24] Nelson C. M. Herron M. J. Felsheim R. F. Schloeder B. R. Grindle S. M. Chavez A. O. (2008). Whole genome transcription profiling of *Anaplasma phagocytophilum* in human and tick host cells by tiling array analysis. BMC Genomics 9:364 10.1186/1471-2164-9-364 18671858 PMC2527338

[B25] Noh S. M. Ueti M. W. Palmer G. H. Munderloh U. G. Felsheim R. F. Brayton K. A. (2011). Stability and tick transmission phenotype of gfp-transformed *Anaplasma marginale* through a complete *in vivo* infection cycle. Appl. Environ. Microbiol. 77, 330–334 10.1128/AEM.02096-10 21057014 PMC3019711

[B26] Pierle S. A. Hammac G. K. Palmer G. H. Brayton K. A. (2013). Transcriptional pathways associated with the slow growth phenotype of transformed *Anaplasma marginale*. BMC Genomics 14:272 10.1186/1471-2164-14-272 23607288 PMC3646689

[B27] Postigo M. Taoufik A. Bell-Sakyi L. Bekker C. P. de Vries E. Morrison W. I. (2008). Host cell-specific protein expression *in vitro* in *Ehrlichia ruminantium*. Vet. Microbiol. 128, 136–147 10.1016/j.vetmic.2007.09.023 18006251

[B28] Pruneau L. Emboule L. Gely P. Marcelino I. Mari B. Pinarello V. (2012). Global gene expression profiling of *Ehrlichia ruminantium* at different stages of development. FEMS Immunol. Med. Microbiol. 64, 66–73 10.1111/f574-695X.2011.00901.x 22098128

[B29] Rikihisa Y. Lin M. (2010). *Anaplasma phagocytophilum* and *Ehrlichia chaffeensis* type IV secretion and Ank proteins. Curr. Opin. Microbiol. 13, 59–66 10.1016/j.mib.2009.12.008 20053580 PMC3251840

[B30] Seo G. M. Cheng C. Tomich J. Ganta R. R. (2008). Total, membrane, and immunogenic proteomes of macrophage- and tick cell-derived *Ehrlichia chaffeensis* evaluated by liquid chromatography-tandem mass spectrometry and MALDI-TOF methods. Infect. Immun. 76, 4823–4832 10.1128/IAI.00484-08 18710870 PMC2573352

[B31] Stich R. W. Kocan K. M. Palmer G. H. Ewing S. A. Hair J. A. Barron S. J. (1989). Transstadial and attempted transovarial transmission of *Anaplasma marginale* by *Dermacentor variabilis*. Am. J. Vet. Res. 50, 1377–1380 2782719

[B32] Troese M. J. Kahlon A. Ragland S. A. Ottens A. K. Ojogun N. Nelson K. T. (2011). Proteomic analysis of *Anaplasma phagocytophilum* during infection of human myeloid cells identifies a protein that is pronouncedly upregulated on the infectious dense-cored cell. Infect. Immun. 79, 4696–4707 10.1128/IAI.05658-11 21844238 PMC3257945

[B33] Vachiéry N. Marcelino I. Martinez D. Lefrancois T. (2013). Opportunities in diagnostic and vaccine approaches to mitigate potential heartwater spreading and impact on the American mainland. Dev. Biol. (Basel.) 135, 191–200 10.1159/000190050 23689897

[B34] Zivkovic Z. Blouin E. F. Manzano-Roman R. Almazán C. Naranjo V. Massung R. F. (2009). *Anaplasma phagocytophilum* and *Anaplasma marginale* elicit different gene expression responses in cultured tick cells. Comp. Funct. Genomics 2009:705034 10.1155/2009/705034 19636428 PMC2712686

[B35] Zivkovic Z. Esteves E. Almazán C. Daffre S. Nijhof A. M. Kocan K. M. (2010). Differential expression of genes in salivary glands of male Rhipicephalus (*Boophilus*) microplus in response to infection with Anaplasma marginale. BMC Genomics 11:186 10.1186/1471-2164-11-186 20298599 PMC2848250

